# Editorial: Rhizosphere conversation among the plant-plant microbiome-soil under consecutive monoculture regimes

**DOI:** 10.3389/fmicb.2022.1061427

**Published:** 2022-10-24

**Authors:** Hongmiao Wu, Changxun Fang, Antonino Malacrinò, Traud Winkelmann, Wu Xiong

**Affiliations:** ^1^College of Resource and Environment, Anhui Agricultural University, Hefei, China; ^2^College of Life Sciences, Fujian Agriculture and Forestry University, Fuzhou, China; ^3^Department of Agriculture, Università degli Studi Mediterranea di Reggio Calabria, Reggio Calabria, Italy; ^4^Institute of Horticultural Production Systems, Leibniz Universität Hannover, Hannover, Germany; ^5^College of Resource and Environment, Nanjing Agricultural University, Nanjing, China

**Keywords:** consecutive monoculture, negative plant-soil feedback, plant microbiome, rhizosphere interactions, rhizosphere management, soil microbiome

Intensive agricultural and horticultural cultivation, including consecutively growing the same crop in the same fields, has been contributing to meet the increasing food demands of a rapid growing human population (Diaz-Ambrona and Maletta, 2014). However, intensive consecutive monoculture results in replant disease/soil sickness, which causes serious plant diseases and subsequent declines in the quality and quantity of crop products (Xiong et al., 2017; Winkelmann et al., 2019; Wu and Lin, 2020; Zhou and Wu, 2021). The current knowledge suggests that the factors underlying replant disease may be associated with soil nutrient imbalance (Huang et al., 2013), accumulation of root exudate autotoxicity (Zhang et al., 2019; Busnena et al., 2021), and changes in the rhizosphere microbial community (Li et al., 2014; Wu et al., 2019; Balbín-Suárez et al., 2021). Yet, we still know little about the mechanisms behind the negative effects of consecutive monoculture regimes on plants, which might hinder the key to develop strategies to alleviate replant disease.

Healthy plants are colonized by a rich diversity of microbes (i.e., bacteria, fungi, protists, and viruses), forming complex microbial consortia that impact plant growth and health. Increasing evidence is showing that the accumulation of soil-borne pathogens (e.g., *Fusarium, Pythium, Alternaria, Ralstonia*, members of *Nectriaceae*) at the expense of plant-beneficial microbes (e.g., *Pseudomonas, Bacillus, Paenibacillus*) might be a major driving factor of replanting disease (Lareen et al., 2016; Xiong et al., 2017; Yim et al., 2017; Popp et al., 2020; Wu and Lin, 2020). For example, the consecutive monoculture regimes significantly increased the abundance of potential pathogenic *Ralstonia* sp. and *Fusarium oxysporum* in the plant leaf and root of *Radix pseudostellariae* (Wu et al., 2022b), increased the diversity of soil-borne plant viruses in the rhizosphere of *R. pseudostellariae* (Wu et al., 2022a), and caused the strong accumulation of plant parasites, plant pathogens, and parasites while significantly reducing the relative abundance of bacteria-feeders nematodes and omnivores (Wu et al., 2021). The interactions between plant, soil, and microorganisms within the soil food-web play a crucial role in creating the conditions that cause the negative effects due to consecutive monoculture. Previous research mainly focused on changes in the soil microbial community structure and composition under consecutive monoculture conditions (Wu et al., 2021), while the effects of intensive monoculture on the overall plant and soil microbiome, in combination with the role determined by root exudates, received little attention.

As a response to the importance of plant-plant microbiome-soil interactions in replant disease, we proposed the Research Topic “Rhizosphere conversation among the Plant-Plant Microbiome-Soil under Consecutive Monoculture Regimes.” In this Research Topic, we have collected six original research and one review articles that contribute on expanding our knowledge about the rhizosphere ecological processes under consecutive monoculture regimes. In their review, Somera and Mazzola comprehensively focused on the multiple factors that contribute to generate an apple replant disease-suppressive soil microbiome and highlighted the importance of considering host genetic factors. Reim et al. contributed to the understanding of apple plant response to replant disease by comparing the transcriptome of two apple genotypes differing in susceptibility in conducive and sterilized soil. Yuan et al. showed that the plant *Pseudostellaria heterophylla* is able to recruit plant-beneficial microbes against the pathogen *Fusarium oxysporum* under continuous monocropping regime. Cui et al. found a more pronounced effect driven by continuous cropping of sugar beet on the fungal than on the bacterial communities inhabiting different plant compartments (bulk soil, rhizosphere soil, and beetroot). Pang et al. suggested that the sugarcane–peanut intercropping pattern could potentially improve soil nutrients, cane agronomic parameters, peanut yield, and bacteria diversity in sugarcane root systems compared to the monoculture farming system. Similarly, Bai et al. found that intercropping walnut and tea positively impacted the soil's nutritional conditions and helped in enriching soil with beneficial bacterial and fungal taxa, suggesting that intercropping was able to alleviate the replant disease by altering the plant-associated microbial communities. He et al. studied the response mechanism of alien invasive and the native plants to acid rain by analyzing plant phenotypic characteristics, soil physicochemical properties, and rhizosphere microbial communities.

Overall, the papers in this Research Topic focus on plant health and reveal the responses of soil physicochemical properties, plant characteristics and soil microbial community to environmental conditions generated by consecutive monoculture regimes. Plant health is intimately influenced by the plant associated microbiome, a complex assembly of organisms that changes dramatically throughout plant development (Xiong et al., 2020). The recruitment of microorganisms in the rhizosphere occurs *via* root exudates directed from plants to microorganisms, and subsequent interactions between microorganisms and between microorganisms and the host plant (Doornbos et al., 2012; Sasse et al., 2018; Balbín-Suárez et al., 2021). Previous studies showed the rhizosphere protists within the microbiome to be key determinants of plant health (Geisen et al., 2018; Xiong et al., 2020) and the plant microbiome and that they are able to alter plant metabolic functions and enhance the disease resistance to pathogens (Chaudhry et al., 2021). Moreover, soil-borne plant viruses can potentially infect plants through mechanical friction, nematode vectors and fungal vectors (Reavy et al., 2014; Wu et al., 2022a). While the study of the consequences of plant-microbiome-environment interactions is attracting a wide interest, we are only at the beginning of understanding the mechanisms that regulate the plant responses to their own interactions with the community of microorganisms they co-inhabited as well as higher order soil organisms. We are optimistic that soon we will be able to combine the power of sequencing technologies, high-throughput phenotyping, high-performance computing, and big data approaches coupled to machine learning to understand the rules that regulate plant-microbiome interactions, and how to exploit them to support a sustainable agriculture.

## Author contributions

HW drafted the editorial. All authors contributed to editorial revision and approved the final paper.

## Funding

This work was partially supported by the National Natural Science Foundation of China (82003884), University Natural Science Research Project of Anhui Province (KJ2021A0137), and the High-Level Scientific Research Foundation for the introduction of talent (rc522103).

## Conflict of interest

The authors declare that the research was conducted in the absence of any commercial or financial relationships that could be construed as a potential conflict of interest.

## Publisher's note

All claims expressed in this article are solely those of the authors and do not necessarily represent those of their affiliated organizations, or those of the publisher, the editors and the reviewers. Any product that may be evaluated in this article, or claim that may be made by its manufacturer, is not guaranteed or endorsed by the publisher.

## References

[B1] Balbín-Suárez A. Jacquiod S. Rohr A. D. Liu B. Flachowsky H. Winkelmann T.. (2021). Root exposure to apple replant disease soil triggers local defense response and rhizoplane microbiome dysbiosis. FEMS Microbiol. Ecol. 97, fiab031. 10.1093/femsec/fiab031 33587112

[B2] Busnena B. A. Beuerle T. Mahnkopp-Dirks F. Winkelmann T. Beerhues L. Liu B.. (2021). Formation and exudation of biphenyl and dibenzofuran phytoalexins by roots of the apple rootstock M26 grown in apple replant disease soil. Phytochemistry 192, 112972. 10.1016/j.phytochem.2021.112972 34624729

[B3] Chaudhry V. Runge P. Sengupta P. Doehlemann G. Parker J. E. Kemen E.. (2021). Shaping the leaf microbiota: plant-microbe-microbe interactions. J. Experi. Botany 72, 36–56. 10.1093/jxb/eraa417 32910810 PMC8210630

[B4] Diaz-Ambrona C. G. H. Maletta E. (2014). Achieving global food security through sustainable development of agriculture and food systems with regard to nutrients, soil, land, and waste management. Curr. Sustain. Renew. Energy Rep. 1, 57–65. 10.1007/s40518-014-0009-2

[B5] Doornbos R. F. van Loon L. C. Bakker P. A. H. M. (2012). Impact of root exudates and plant defense signaling on bacterial communities in the rhizosphere. A review. Agron. Sustain. Develop. 32, 227–243. 10.1007/s13593-011-0028-y

[B6] Geisen S. Mitchell E. A. D. Adl S. Bonkowski M. Dunthorn M. Ekelund F.. (2018). Soil protists: a fertile frontier in soil biology research. FEMS Microbiol. Rev. 42, 293–323. 10.1093/femsre/fuy006 29447350

[B7] Huang L. F. Song L. X. Xia X. J. Mao W. H. Shi K. Zhou Y. H.. (2013). Plant-soil feedbacks and soil sickness: from mechanisms to application in agriculture. J. Chem. Ecol. 39, 232–242. 10.1007/s10886-013-0244-9 23385367

[B8] Lareen A. Burton F. Schafer P. (2016). Plant root-microbe communication in shaping root microbiomes. Plant Molec. Biol. 90, 575–587. 10.1007/s11103-015-0417-8 26729479 PMC4819777

[B9] Li X. Ding C. Hua K. Zhang T. Zhang Y. Zhao L.. (2014). Soil sickness of peanuts is attributable to modifications in soil microbes induced by peanut root exudates rather than to direct allelopathy. Soil Biol. Biochem. 78, 149–159. 10.1016/j.soilbio.2014.07.019

[B10] Popp C. Wamhoff D. Winkelmann T. Maiss E. Grunewaldt-Stöcker G. (2020). Molecular identification of Nectriaceae in infections of apple replant disease affected roots collected by Harris Uni-Core punching or laser microdissection. J. Plant Dis. Protect. 127, 571–582. 10.1007/s41348-020-00333-x

[B11] Reavy B. Swanson M. M. Taliansky M. (2014). “Viruses in Soil,” in Interactions in Soil: Promoting Plant Growth, eds J. Dighton and J.A. Krumins, (Netherlands, Dordrecht: Springer), 163–180. 10.1007/978-94-017-8890-8_8

[B12] Sasse J. Martinoia E. Northen T. (2018). Feed your friends: do plant exudates shape the root microbiome? Trends Plant Sci. 23, 25–41. 10.1016/j.tplants.2017.09.003 29050989

[B13] Winkelmann T. Smalla K. Amelung W. Baab G. Grunewaldt-Stöcker G. Kanfra X.. (2019). Apple replant disease: causes and mitigation strategies. Curr Issues Molec. Biol. 30, 89–106. 10.21775/cimb.030.089 30070653

[B14] Wu H. Lin W. (2020). A commentary and development perspective on the consecutive monoculture problems of medicinal plants. Chin. J. Eco-Agric. 28, 775–793. 10.13930/j.cnki.cjea.190760

[B15] Wu H. Qin X. Wang J. Wu L. Chen J. Fan J.. (2019). Rhizosphere responses to environmental conditions in Radix pseudostellariae under continuous monoculture regimes. Agriculture, Ecosystems and Environment 270, 19–31. 10.1016/j.agee.2018.10.014

[B16] Wu H. Wu H. Qin X. Lin M. Zhao Y. Rensing C.. (2021). Replanting disease alters the faunal community composition and diversity in the rhizosphere soil of Radix pseudostellariae. Agric. Ecosyst. Environ. 310, 1–7. 10.1016/j.agee.2021.107304

[B17] Wu H. Yan W. Wu H. Zhang J. Zhang Z. Zhang Z.. (2022a). Consecutive monoculture regimes differently affected the diversity of the rhizosphere soil viral community and accumulated soil-borne plant viruses. Agric. Ecosyst. Environ. 337, 108076. 10.1016/j.agee.2022.108076

[B18] Wu H. Zhang Z. Wang J. Qin X. Chen J. Wu L.. (2022b). Bio-fertilizer amendment alleviates the replanting disease under consecutive monoculture regimes by reshaping leaf and root microbiome. Microbial. Ecol. 84, 452–464. 10.1007/s00248-021-01861-1 34554283

[B19] Xiong W. Guo S. Jousset A. Zhao Q. Wu H. Li R.. (2017). Bio-fertilizer application induces soil suppressiveness against Fusarium wilt disease by reshaping the soil microbiome. Soil Biol. Biochem. 114, 238–247. 10.1016/j.soilbio.2017.07.016

[B20] Xiong W. Song Y. Q. Yang K. M. Gu Y. Wei Z. Kowalchuk G. A.. (2020). Rhizosphere protists are key determinants of plant health. Microbiome 8, 1–9. 10.1186/s40168-020-00799-9 32127034 PMC7055055

[B21] Yim B. Nitt H. Wrede A. Jacquiod S. Sørensen S. J. Winkelmann T.. (2017). Effects of soil pre-treatment with Basamid^®^ Granules, *Brassica juncea, Raphanus sativus*, and *Tagetes patula* on bacterial and fungal communities at two apple replant disease sites. *Front*. Microbiol. 8, 1604. 10.3389/fmicb.2017.01604 28919882 PMC5586068

[B22] Zhang B. Weston P. A. Gu L. Zhang B. Li M. Wang F.. (2019). Identification of phytotoxic metabolites released from Rehmannia glutinosa suggest their importance in the formation of its replant problem. Plant Soil 441, 439–454. 10.1007/s11104-019-04136-4

[B23] Zhou X. Wu F. (2021). Land-use conversion from open field to greenhouse cultivation differently affected the diversities and assembly processes of soil abundant and rare fungal communities. Sci. Total Environ. 788, 147751. 10.1016/j.scitotenv.2021.147751 34023613

